# Investigation of Novel Flax Fiber/Epoxy Composites with Increased Biobased Content

**DOI:** 10.3390/polym15194030

**Published:** 2023-10-09

**Authors:** Bianca Dal Pont, Vito Gigante, Luca Panariello, Ilaria Canesi, Laura Aliotta, Andrea Lazzeri

**Affiliations:** 1 Department of Civil and Industrial Engineering, University of Pisa, Via Diotisalvi, 2, 56122 Pisa, Italy; bianca.dalpont@ing.unipi.it (B.D.P.); luca.panariello@ing.unipi.it (L.P.); andrea.lazzeri@unipi.it (A.L.); 2 Interuniversity National Consortium of Materials Science and Technology (INSTM), Via Giusti 9, 50121 Florence, Italy; 3 Planet Bioplastics, Via San Giovanni Bosco 23, 56127 Pisa, Italy; ilariacanesi@planetbioplastics.com

**Keywords:** fibers, mechanical properties, thermosets

## Abstract

Currently, biobased epoxy resins derived from plant oils and natural fibers are available on the market and are a promising substitute for fossil-based products. The purpose of this work is to investigate novel lightweight thermoset fiber-reinforced composites with extremely high biobased content. Paying attention to the biobased content, following a cascade pathway, many trials were carried out with different types of resins and hardeners to select the best ones. The most promising formulations were then used to produce flax fiber reinforced composites by vacuum bagging process. The main biocomposite properties such as tensile, bending, and impact properties as well as the individuation of their glass transition temperatures (by DSC) were assessed. Three biocomposite systems were investigated with biobased content ranging from 60 to 91%, obtaining an elastic modulus that varied from 2.7 to 6.3 GPa, a flexural strength from 23 to 108.5 MPa, and Charpy impact strength from 11.9 to 12.2 kJ/m^2^. The properties reached by the new biocomposites are very encouraging; in fact, their stiffness vs. lightweight (calculated by the *E/ρ*^3^ ratio) is comparable to some typical epoxy–glass composites.

## 1. Introduction

Composites represent an extensive and significant category of materials, with an annual global production exceeding 10 million tons. Over the past decade, the composite market has experienced growth at a rate of 5–10% per year [1]. The relevance of composite materials has become crucial in every manufacturing sector [2]. However, environmental concerns have pushed industrial interests towards natural-based materials [3]. Natural fiber-reinforced composites (NFRC) have gained importance in recent years, and there is constant research in enhancing their biobased content without losing thermal and mechanical properties [4]. In this context, the adoption of natural fibers offers several advantages, including cost-effectiveness, biodegradability, reduced processing equipment wear, and more energy-efficient production processes (approximately 45% lower energy consumption) [5]. Energy recovery and carbon dioxide neutrality are additional features for low environmental impact that are ensuring a broad use of natural fibers [6,7]. Consequently, NFRCs have found successful applications in automotive and aircraft components, sports equipment, and electrical parts due to their environmental friendliness, lightweight nature, commendable mechanical behavior, and sound attenuation [8,9,10]. In the last decade, a great number of scientific articles have been dedicated to the investigation of innovative NFRC with several natural fibers [11,12,13,14,15,16,17,18]. Encouraging results in this field have been achieved, and some NFRC systems reached equivalent strength and stiffness levels to glass fiber-reinforced composites (GFRC); however, an increment of the fiber volume fraction was necessary, which also led to a composite biobased content enhancement [19]. Steps towards more sustainable NFRC have also been made by Bharath et al. [20] that successfully obtained a composite laminate using natural rice husk and epoxy resin without using environmental unfriendly chemical solvents. However, to further increase the NFRC sustainability only the substitution of natural fibers for synthetic ones is not sufficient. It is therefore necessary to investigate more deeply the substitution of fossil-based thermoset matrices with biobased ones. Therefore, research for new technological and eco-friendly solutions for the most common thermosets matrices such as epoxy, phenolic, and polyester resins is of pivotal interest for the composites industry [21,22].

In particular, epoxy resins are the most common thermoset matrices for which several works may be found where epoxies have been coupled with different natural fibers [23,24,25,26]. Epoxies are obtained mainly from bisphenol A diglyceryl ether (DGEBA), one of the most highly adopted resins produced by epichlorohydrin and bisphenol A. These materials have elevated stiffness, stress at break, and hardness coupled with good chemical, corrosion, and moisture resistance and a good thermal adhesivity [27]; however, they represent a negative factor in the environmental balance because of their fossil-based nature. Consequently, the use of biobased resins to replace traditional epoxy resin is a necessary path to follow for sustainability. The most promising biobased building blocks are represented by vegetable oils, and the main products are available on the market. The abundance of C=C unsaturation in vegetable oils is optimal for structuring thermosetting resins since the double bond provides reactive groups that can be easily epoxidized [28]. Resins from vegetal oils that show excellent potential in replacing fossil-based counterparts can be represented by epoxidized soybean oil (ESO) or epoxidized linseed oil (ELO) and their derivatives [29,30,31]. However, ESO/ELO-based composites cannot yet fully compete with high-performance composites which have superior tensile and flexural strengths. Moreover, the aromatic structure of polyphenolic resins plays a fundamental role, conferring to this class of resins better chemical and thermal resistance, as well as higher mechanical resistance in respect of aliphatic resin from vegetable oils [32]. In this context, promising biobased resins arise from tannins and cardanol. The latter, sourced from cashew nutshell oil, represents a non-edible by-product. Cardanol is the major component of cashew nutshell liquid (CNSL), and it is a foundational building block for biobased polymers. Cardanol is commercial available with di- and poly-epoxy monomers derived from cardanol through a two-step reaction [33,34,35].

Among commercially available partially biobased epoxy resins [36], the most promising products are the cardanol-based resins produced by Cardolite^®^ and the sustainable GreenPoxy solutions offered by Sicomin^®^ resins. Their biobased content in combination with their low viscosity and high epoxy functionality makes them very attractive. Very few works have been published that have been carried out with these resins. Some studies have been undertaken with the resins produced by Sicomin^®^. For example, Torres-Arellano et al. [37] developed bio-laminates through the vacuum-assisted resin infusion process using natural fibers (such as Henequen and Jute) as reinforcing fabrics, obtaining high values of composites stiffness and strength. Saidane et al. [38], instead, studied the effect of the temperature variation and the stacking sequence on the internal stresses of GreenPoxy/flax fibers composites. Moreover, Sala et al. [39] investigated the influence of hygrothermal conditions on the creep/recovery behavior of three high-grade composites made of GreenPoxy and flax and hemp fibers. Although there are not many studies on GreenPoxy based composites, research on this resin has started. On the other hand, no published studies exist on composites containing the epoxy resins produced by Cardolite^®^.

The aim of this work is to investigate new biocomposite systems to increase the biobased content of laminated composites so as to achieve thermo-mechanical properties comparable to the currently glass-reinforced, petro-based solutions. To reach this final goal, optimal mixing of commercial biobased epoxy resins (GreenPoxy and Cardolite) was undertaken for the first time to select the best resin formulation with high biobased content and mechanical properties. Subsequently, composite laminates production, by vacuum bagging process, was carried out by adding twill flax fibers that were chosen for their strength-to-weight ratio, comparable to E-glass and aramid ones [40,41,42,43]. The novel NFRC were investigated from mechanical, thermal, and morphological points of view [44]. The results obtained are of novelty because to the best of our knowledge, no published studies exist where the final composite biobased content achieved is so high. Moreover, this work lays the basis for further investigations better to tailor the biocomposite mechanical properties with respect to the final biobased content achievable. Moreover, the encouraging results obtained represent a great opportunity to reduce the environmental impact of composites with minimal product and process modifications.

## 2. Materials and Methods

### 2.1. Materials

The materials used in this work are listed below:

#### 2.1.1. Resins

EPIKOTE Resin 828LVEL, provided by Hexion (Columbus, OH, USA): this is a medium-viscosity liquid epoxy resin produced from bisphenol A resin and epichlorohydrin containing no diluent. According to the producer’s datasheet, Epikote 828 provides good pigment wetting and good resistance to filler settling along with a high level of mechanical and chemical resistance properties in a cured state (density 25 °C: 1.16 g/cm^3^; viscosity: 10,000–12,000 mPa·s).

NC-547, provided by Cardolite (Gent, Belgium): this is a biobased polyglycidyl ether of an alkenyl phenol formaldehyde novolac resin. The biobased content is about 84%, as declared by the producer (density 25 °C: 0.935 g/cm^3^; viscosity: 20,000–50,000 mPa∙s).

SR GREENPOXY 56, provided by Sicomin (Châteauneuf-les-Martigues, France): this is a low-viscosity epoxy resin composed of a mix of bisphenol and epichlorohydrin obtained from the use of oleaginous oil. The biobased content is about 56%, as declared by the producer (density: 1.198 g/cm^3^; viscosity: 800 mPa∙s).

#### 2.1.2. Curing Agents

SX8-EVOB purchased from Mates Italiana (Milano, Italy): this is a petro-based low-viscosity curing agent based on modified cycloaliphatic polyamine. The producer suggests a mixing ratio with hardener of 100:30 by weight for a resin with EEW of 800.

NC-541 provided by Cardolite (Gent, Belgium): this is a solvent-free phenalkamine curing agent with excellent rapid cure properties, even at low temperatures. The bio content of this hardener is 82%, as declared by the producer. The producer suggests a mixing ratio with hardener of 100:68 by weight for a resin with EEW of 190.

SD-4771 provided by Sicomin (Châteauneuf-les-Martigues, France): this is a petro-based hardener with slow reactivity that grants a good flow during the infusion process. It is mainly composed from the reaction product of di-tri and tetra propoxylated propane-1,2 diol with ammonia. The producers suggest curing at room temperature with a post cure between 40 and 80 °C. They suggest a mixing ratio with hardener of 100:30 by weight for a resin with EEW of 800.

The resins were mixed appropriately with different curing agents considering the mechanical properties declared in the datasheet and paying attention to the final biobased content. The three different formulations investigated in this work are listed in Table 1.

#### 2.1.3. Fibers

Flax fibers FLAX DRY BL300, TWILL, provided by Mascherpa (Milano, Italy): this flax twill has a smooth surface texture and is suitable to be used as surface or backing plies. The roll width is 1000 mm, and the thickness is 0.3 mm (grammage: 300 g/m^2^; density: 1.29 g/cm^3^; tensile strength in warp: 113 MPa).

### 2.2. Composite Formulations

For composite production, the same resin mix described in Table 1 was used, adding 30% vol. of twill flax fibers. The composite names corresponding to the different resin mixes are given in Table 2. A 50:50 mix between the Epikote petro-based resin and biobased Cardolite resin was used for MT1-FT composite; a 50:50 mix between biobased resins (Cardolite and GreenPoxy) was adopted for MB1-FT composite while only GreenPoxy constituted BB1-FT composite.

### 2.3. Evaluation of the Biobased Content

For both resins and composites, the biobased content was evaluated as follows:

(1) $$Resins\;Biobased\;content=\left[{\%bio}_{resin}\cdot \frac{w_{r}}{w_{m}}\right]+\left[{\%bio}_{hardener}\cdot \frac{w_{h}}{w_{m}}\right]$$

(2) $$Composites\;Biobased\;content=\left[{\%bio}_{matrix}\cdot \frac{w_{m}}{w_{c}}\right]+\left[{\%bio}_{fibre}\cdot \frac{w_{f}}{w_{c}}\right]$$

where w_r_ is the weight in grams of the resin, w_h_ is the weight of the hardener, w_m_ is the weight of the matrix system (resin + hardener), w_f_ is the weight of the fiber, and w_c_ is the weight of the composite.

### 2.4. Infusion

#### 2.4.1. Viscosity Measurement

Viscosity measurements were performed using an RT-100 plus viscometer (Lamy Rheology Instruments, Champagne au Mont d’Or, France) equipped with an MK-SV27 spindle thermocell viscosity measuring unit. The crucible was filled with approximately 12 mL of resin. Viscosity measurements were performed at 25 °C. Testing time was 60 s while the shear rate was adjusted to 20 s^−1^, as suggested by Govignon et al. [45]. Five measurements for each type of formulation were acquired. Both resin and resin-hardener system viscosity were evaluated.

#### 2.4.2. Vacuum Bagging

**Figure 1:**
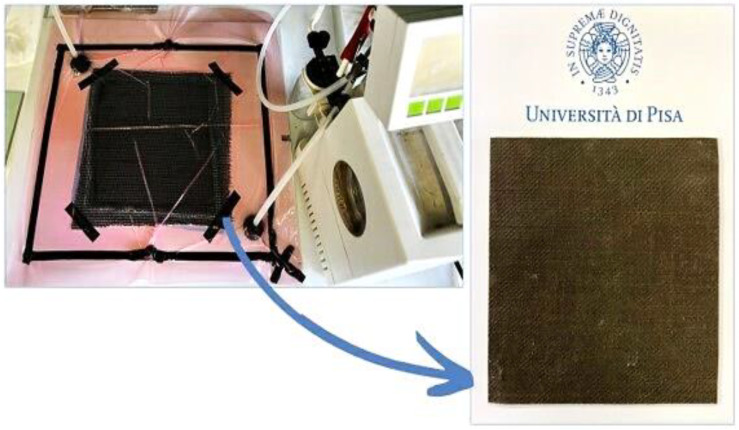
Vacuum bagging process and one of the flax fiber-laminated composites produced.

Composites were produced through the vacuum bagging technique as shown in Figure 1 that, in respect of other composite manufacturing techniques, allows the production of high-quality and high-performance composites [46]. The mold plate used for composite manufacturing was a flat surface of 500 mm × 500 mm. The process of composite manufacturing was divided into different stages: preparation of plies, preparation of mold plate, stacking of reinforcement, resin mixing, vacuum, infusion, and curing. Fiber woven layers were cut to a 200 × 200 mm size and fixed with tape to prevent fraying. It has been written [47,48] that heat treatments should be used to reduce the amount of moisture in flax fiber, however high temperature and long periods of time decrease the mechanical properties of the fibers. For that reason, a 1 h cycle at 110 °C was carried out to avoid fiber damage without decreasing their strength.

Peel ply, microperforated ply, and drainage ply were cut to a 220 × 220 mm size. The vacuum bag was cut to a 400 × 400 mm size, to avoid vacuum leakage. The mold was well cleaned to remove dust and residues of previous vacuum bagging. The double-side tape was positioned on the mold in a square shape with a dimension of 350 × 350 mm size. The mold was then coated with a release agent (T16 Hi-temp mold release paste wax, TR, South Gate, CA, USA). The release agent was applied to ensure that the laminate conveniently separated from the mold after curing. Layers were placed on the plate following a specific sequence: stacked fibers, peel ply, microperforated ply, drainage ply, and vacuum bag. Two “valves” were placed with double-sided tape at the ends of the tissues along with a spiral tube (this helps to distribute the resin flow homogeneously).

**Figure 2:**
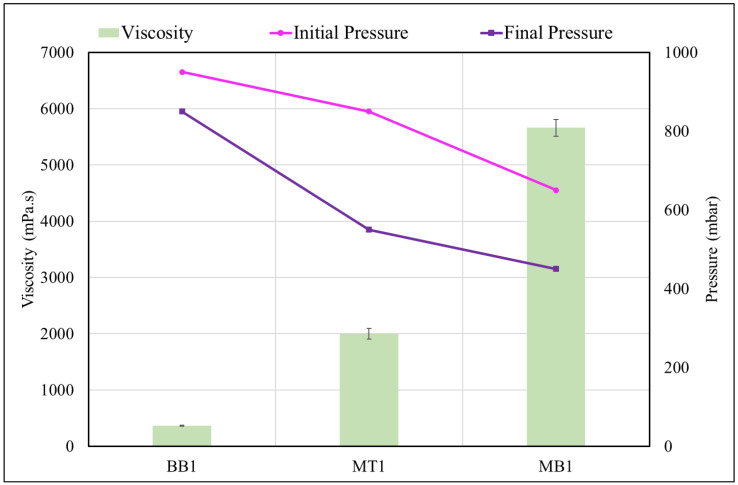
Correlation between viscosity and pressure.

The vacuum bag was placed and secured well to the tape to avoid any folding. Two vacuum hoses (inlet and outlet) were connected to the valves. Initially, the outlet pipe was connected to the vacuum pump, while the inlet hose was clamped ensuring that no air could enter. The pump was turned on until the vacuum reached the desired value. Once the vacuum remained stable, the inlet tube was placed inside the beaker containing the resin. To guarantee a correct fiber/matrix ratio, the infusion pressure value had to be set to ensure constant resin flow through the woven, avoiding an excessive reduction of fiber volume. In Figure 2, it is possible to see the initial/final pressure and the infusion temperature of each resin formulation. Once the inlet tube was placed in the resin, it began to flow and the infusion progression could be easily evaluated by sight. The infusion time and pressure used depended on the viscosity of the resin; however, with all the fibers, the process condition was optimized according to the corresponding resin. It is evident that the viscosities were adequate to facilitate an optimal infusion of the matrix into the flax fibers, particularly in the case of BB1, which exhibited the lowest viscosity value without initiating crosslinking. On the other hand, MB1 demonstrated a value approximately fivefold greater than BB1. This divergence was mirrored by the corresponding pressure values employed to achieve proper infusion. Both viscosity and pressure were meticulously optimized to ensure thorough impregnation of the fibers, thereby averting void formation and ensuring reproducibility of the end product.

When the infusion was completed, both tubes were clamped. The mold plates with the prepared composite were cured in a laboratory oven following the curing cycle (24 h T_room_ + 4 h at 60 °C).

#### 2.4.3. Laminate Layering

The way of arranging the different layers of reinforcing material in a laminate is known as the stacking sequence. The fibers were oriented in a different way to minimize anisotropy in the final properties of composites. Equation (3) [49] was used to determine the layers to be included in the composite:

(3) $$d=\frac{nA_{w}}{{\rho_{f}V}_{f}}$$

where “d” is the thickness, “n” is the number of plies, “A_w_” is the grammage, “ρ_f_” and “V_f_” are the density and the volume fraction of the fibers, respectively. Therefore, by knowing the fiber density, keeping the fiber volume fraction fixed at 30%, and setting a thickness of at least 2.5 mm, it was possible to calculate the number of required plies. Therefore, there should be 4 plies for twill flax woven.

### 2.5. Composites Characterization

#### 2.5.1. Tensile Test

The tensile properties of the composites were evaluated according to ASTM D3039. The specimens were rectangular with 80 × 110 mm dimension. For the mechanical characterization, at least 5 specimens were tested. Specimens were preconditioned at 50%RH for 2 days before the test. Tensile tests were carried out on a universal testing machine MTS Criterion model 43 universal tensile (MTS Systems Corporation, Eden Prairie, MN, USA) testing machine, equipped with a 10 kN cell load at a crosshead speed of 2 mm/min, for the initial 0.8 mm of elongation with extensometer, and at 10 mm/min without extensometer for elongation values over 0.8 mm. Tabs are pivotal during unidirectional materials testing against the failure in the gauges. Tabs may also be required when testing unidirectional materials in the matrix direction to prevent gripping damage [50]. The most consistently used bonded tab material is continuous E-glass fiber-reinforced polymer matrix materials [51]. All composite laminates were tabbed before tensile testing. Composites were initially polished to have a rough surface, which assists in the proper adhesion of tabs with laminates. Elan-tech AW-46 (Elantas, Parma, Italy) was used to stick tabs to laminates. A 3 h cure cycle at 50 °C was carried out to assure adhesive strength.

#### 2.5.2. Flexural Test

The flexural properties of the composites were evaluated according to ASTM D790. A three-point bending test was carried out on a universal testing machine MTS Criterion model 43 universal tensile testing machine (MTS Systems Corporation, Eden Prairie, MN, USA), equipped with a 10 kN cell load at a crosshead speed of 2 mm/min [52]. A bar of rectangular cross section rests on two supports and is loaded by means of a loading nose midway between the supports [53]. A support span-to-depth ratio of 16:1 or 20:1 should be used. The characteristics of composite laminates of this work were carried out with a 20:1 ratio. The specimen with a thickness > 3.2 mm should have 12.7 mm in width. The specimen is deflected until rupture occurs in the outer surface of the test specimen. At least five samples for each formulation were tested, and the mean flexural modulus value was reported. The flexural stress and the flexural modulus were extrapolated from the data [54]. When homogeneous elastic material undergoes a flexural test, the maximum stress in the outer surface of the test specimen occurs at the midpoint. This stress may be calculated for any point on the load–deflection curve by means of the Equation (4):

(4) $$\sigma =\frac{3PL}{2bd^{2}}$$

where the stress in the outer fibers at midpoint is σ (MPa), the load at a given point on the load–deflection curve is P (N), the support span is L (mm), the width of the specimen is b (mm), and the depth of the specimen is d (mm). The flexural modulus was calculated from stress–strain curves using Equation (5):

(5) $$E=\frac{L^{3}m}{4bd^{3}}$$

where the modulus of elasticity in bending is E (MPa), the support span is L (mm), the width of the specimen is b (mm), the depth of the specimen is d (mm), and the angular coefficient of the linear elastic part of the load–deflection curve is m $\left(\frac{N}{mm}\right)$.

#### 2.5.3. Charpy Impact Test

The impact properties of the composites were evaluated according to ISO179. The Charpy test was carried out on a Instron CEAST 9050 machine (INSTRON, Canton, MA, USA) equipped with a 15 J Charpy pendulum. The hammer fall speed of the tests is 4.08 m/s. The specimens were V-notched in the middle by a V-notch manual cutter: notch dimension was 2 mm at 45°. At least 5 specimens were tested, and an average value was reported. The Charpy impact test measures the energy absorbed by a standard notched specimen while breaking under an impact load. This test consists of striking a specimen with a hammer on a pendulum arm while the specimen is attached to the ends. The hammer strikes in the opposite direction of the notch. The energy absorbed by the specimen is accurately determined by measuring the decrease in movement of the pendulum arm.

#### 2.5.4. Thermal Analysis

To evaluate the glass transition temperature, the thermal properties of the resins formulation and their composites were investigated by calorimetric analysis Q200 TA–DSC (TA Instruments, New Castle, DE, USA) equipped with an RSC 90 cooling system. Nitrogen was used as inert and purge gas, and it was set at 50 mL/min for all the measurements. The sampling was carried out taking 10–15 mg of material through the composite thickness. The DSC tests were performed on three different parts of the samples; this procedure guaranteed that the samples were homogenous and the mixing of the resin with the hardener was well executed. The thermal cycle was composed by cooling from room temperature to −20 °C at 10 °C/min, 2 min isotherm at −20 °C, and heating to 120 °C at 10 °C/min.

#### 2.5.5. Optical Analysis

Morphological analysis was carried out on an EM-30 scanning electron microscope (SEM) (Coxem Ltd., Daejeon, Republic of Korea). Sample sections were exposed by cryogenic fracture and were placed on a specimen holder using a conductive adhesive. Before microscope scanning, the surface was sputtered (on Edward S150B; BOC Edwards, Burgess Hill, UK) with gold to increase its conductivity. Image-J software (version 1.52) was used to analyze the SEM images and calculate the void content of the composites.

## 3. Results

### 3.1. Resin Mechanical and Thermal Characterization

Table 3 presents an in-depth analysis of the biobased content, along with mechanical and thermal characterization of the resins. A prominent disparity in biobased content calculated according to Equation (1) is discernible. It can be observed that crescent values of biobased content from 32.34% up to 74.86% were reached passing from MT1 to MB1. It is noteworthy that MB1 has the highest biobased content result. In fact, MB1 is a mix between biobased resins (Cardolite and GreenPoxy) that currently has not yet been investigated. Thanks to this mixing ratio (50:50), the biobased content rises to approximately 75%; this biobased content can be further increased, incorporating a greater amount of natural fibers into the final composite formulation.

With attention to the mechanical properties, the use of a biobased resin in conjunction with a biobased hardener (MB1) emerged as the most advantageous compromise between thermo-mechanical properties and biobased content. Concerning the tensile tests of MT1 and MB1, both stress and strain at break are similar, around 12 MPa and 6.5%, respectively. The results obtained are satisfactory to the study since the properties achieved by MB1 (resin with 75% biobased content) are comparable to those of MT1 (resin with 68% of petroleum sources). A big difference between MB1-MT1 and BB1 can be observed indeed; while MB1-MT1 exhibits low elastic modulus and high elongation at break, the behavior of BB1 is mirrored. Thereby MB1-MT1 present good flexibility while BB1 would be better adaptable for structural applications. Therefore, MB1 present a good compromise between sufficient mechanical and thermal properties and the achievement of a significative biobased content. However, it must be considered that the addition of natural fibers will not only allow a further increase in the biobased content of the final composite material but also a marked improvement of the final thermo-mechanical properties. The Charpy Impact Test had been also run on the matrices, but the data had not been inserted in the paper since MT1 and MB1 showed an excessively rubbery nature of the specimens that made the hammer bounce off the specimen. However, the BB1 value was added as the only one in which a Charpy Impact Strength was detected.

**Figure 3:**
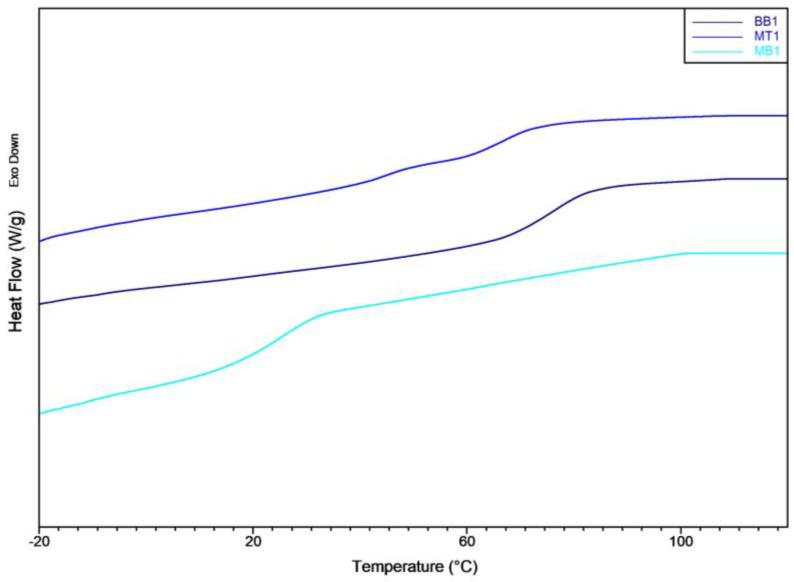
DSC thermogram of the matrix.

A discernible disparity between MT1 and MB1 of about 30 °C in glass transition temperature was noted, Figure 3, signifying that, at the same temperatures, the MB1 resin exhibits better molecular mobility; this is reflected in a reduced elastic modulus.

### 3.2. Composites: Bio Content Analysis, Mechanical-Thermal Characterization and Optical Analysis

#### 3.2.1. Biobased Content

**Figure 4:**
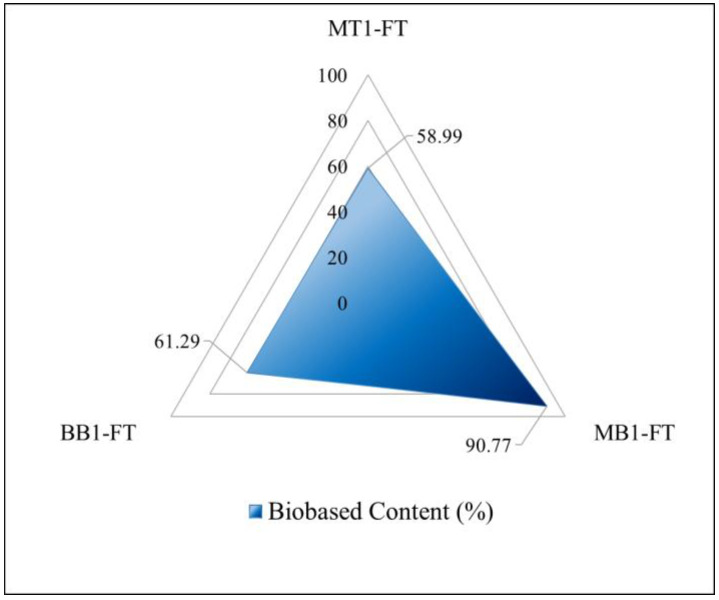
Biobased content radar graphic.

A substantial proportion of biobased constituents was evident across all composite materials, as illustrated in Figure 4. The outcome revealed a noteworthy attainment in terms of biobased content, surpassing 50% in all composites as computed using Equation (2). This advance represents a notable stride within the realm of current NFRC publications and market offerings [55]. Notably, the MT1-FT composite system exhibited a remarkable enhancement of biobased content by +82% in comparison to the MT1 resin, an increase attributed to the integration of flax fibers. Through the incorporation of fibers alongside a partial replacement of the conventional resin (EPIKOTE 828) with a partially biobased variant (NC-547), a notable biobased content of 58.99% was achieved. An innovative substitution strategy was formulated whereby the cardanol resin (NC-547) was subject to partial replacement with the BB1 resin, resulting in a composite designated as MB1-FT. This combination of resins, coupled with flax fibers, yielded the most remarkable outcome: a biobased content of 91%.

#### 3.2.2. Thermo-Mechanical Results

The values obtained from tensile, impact, and DSC tests for the composites are summarized in Table 4.

**Figure 5:**
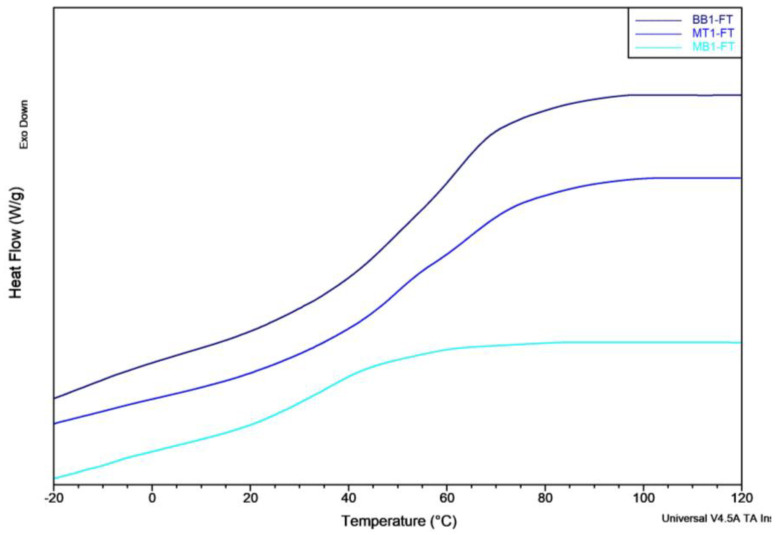
DSC thermogram of composite.

**Figure 6:**
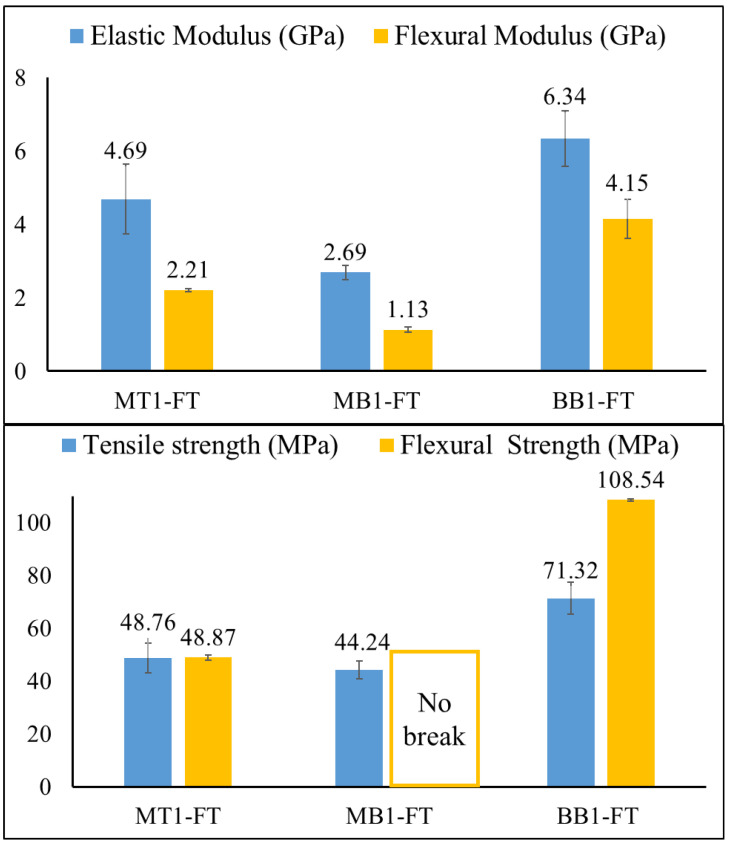
Composite flexural and elastic modulus (**above**) and composites flexural strength (**below**).

All the produced composite materials contained an identical fiber volume fraction of 30%. As could be expected, the addition of natural fibers led to an increase of the composite glass transition temperatures as it can be observed from the DSC thermograms reported in Figure 5. This phenomenon is consistent with what may be found in existing publications regarding other biocomposites and is ascribed to the reduced mobility of resin molecules caused by the presence of natural fibers [56]. Regarding mechanical properties, specifically stiffness, it can be observed that, volume fraction of fiber added being the same, the increment of composites stiffness follows the same order of their corresponding resins formulations. In particular, the MB1-FT possesses the lowest tensile modulus of 2.69 GPa, while BB1-FT has the highest value of 6.34 GPa. In any case, the addition of the flax fibers having high stiffness (around 27.6 GPa [57]) leads to a great increment of all composite stiffness. This trend is also reflected in the flexural modulus, as reported in Figure 6. The highest flexural modulus was achieved by the BB1-FT composite, followed by the MT1-FT composite. The flexural values reached by BB1-FT are interesting, with a value of 108.54 MPa of flexural strength being obtained. The flexural results are comparable to those reported in published works [57,58] regarding other NFRC systems. Conversely, the MB1-FT composite, due to the lower modulus of the resin, its lower T_g_, exhibited the lowest flexural modulus (1.13 GPa). The extreme flexural ductility registered for MB1-FT is also evident when analyzing the flexural strength. In fact, no break was registered during the bending test where the MB1-FT undergoes plastic deformation that overcomes the limit imposed by the flexural normative.

The addition of flax fibers also improves the load bearing capacity of all composites as can be observed from the results of tensile strengths. The increment of tensile strength for all composites may be associated with good adhesion between the matrix and the flax fibers that leads to an efficient load transfer from the matrix to the fiber [59]. The MB1-FT composite is notable, with the highest biobased content, reaching a mechanical strength of about 45 MPa compared to the BB1 matrix tensile strength of 12.20 MPa. The tensile strength of MB1-FT is comparable to that of MT1-FT, while also displaying superior elongation at break and maintaining an elastic modulus below 3 GPa.

However, it must be noted that while BB1-FT has the best tensile and flexural properties, its biobased content reached only 61%. The more rigid starting BB1 matrix makes the composite very stiff. Nevertheless, the excessive stiffness led to a lower Charpy impact resistance. Significantly, the Charpy impact resistance of MB1-FT surpassed that of the other composites. This increment was ascribed to: (i) a stronger interaction between the biobased matrix and the fibers; (ii) the better ductility of the MB1 resin. In particular, the impact resistance of MB1-FT exceeded that of MT1-FT by 61% and that of BB1-FT by 56%.

#### 3.2.3. Optical Analysis

**Figure 7:**
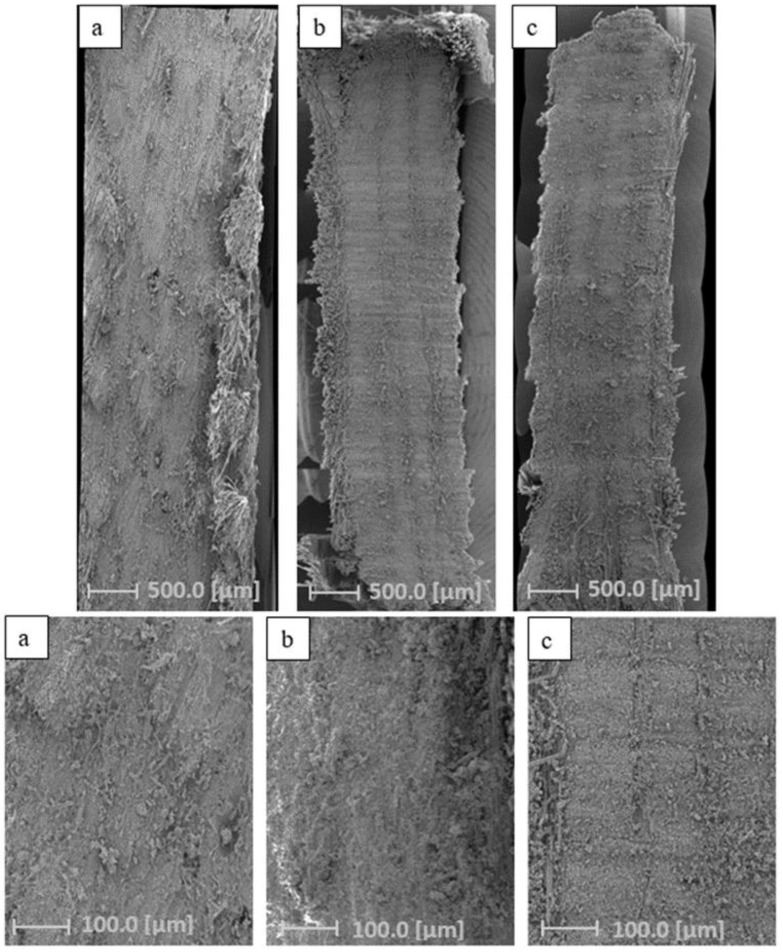
SEM analysis: (**a**) MT1-FT, (**b**) MB1-FT, (**c**) BB1-FT.

To verify that no void formation occurred during the infusion process, sections of composites were examined by SEM; the micrographs are reported in Figure 7.

The stitches of several SEM micrographs were made to better observe any eventual presence of voids within the sample. Results of these analyses provide evidence that in all the composites, no voids were detected, confirming the reproducibility and efficacy of the vacuum bag technique. Good adhesion that was ascribed to a better compatibility with flax fibers for BB1-FT was also confirmed in Figure 7.

#### 3.2.4. Merit Index

The introduction of any kind of reinforcement in a matrix might alter mechanical and thermal properties; therefore, choosing the composition and structure of a composite material for a particular application is not simple. It is also crucial to be aware of any potential changes in the matrix microstructure resulting from the presence of reinforcement. This can be visualized using “Merit Index” maps. The purpose is to evaluate the different combinations offered by the matrices and reinforcements considering the equilibrium among essential properties such as stiffness and lightness.

**Figure 8:**
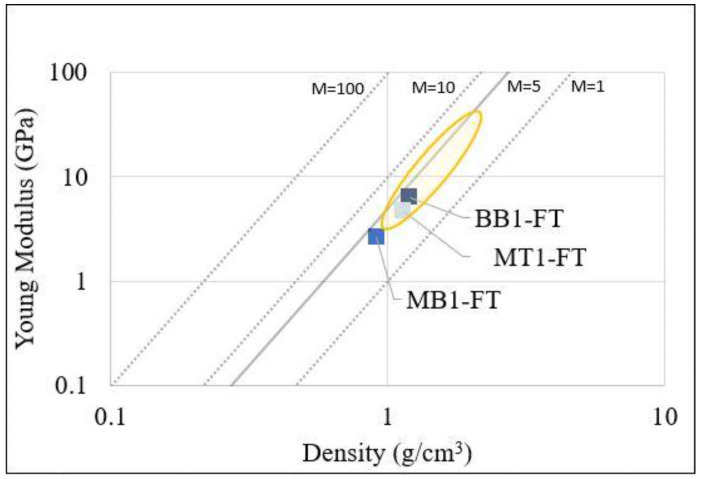
Merit Index of composites.

To better compare different composite systems, a “merit index” for the required performance was calculated. In this context, several models may be applied to individuate the upper and lower bounds on the composite properties involved in the merit index for a given volume fraction of reinforcement [60]. In Figure 8, the dashed lines correspond to constant values of the *E/ρ*^3^ ratio to emphasize the lightness of the material at the same stiffness. This ratio represents the merit indices to be maximized to obtain a minimum component weight consistent with a maximum permissible deflection for different component shapes and loading configurations.

Always in Figure 8, it can be observed that all the composites analyzed in this work remained on the same line, even if the biobased content, the glass transition, and mechanical properties are different, indicating that all of these possessed a similar merit index. Based on their densities, in fact, they demonstrated an adequate stiffness and a merit index comparable to typical epoxy–glass composites, indeed in the yellow circle it can be seen the typical region of merit index where glass fibers reinforced epoxy composites lie [61].

## 4. Conclusions

In this study, the replacement of traditional petro-based resin with biobased ones was investigated to obtain a novel NFRC system with high biobased content. A proper selection and mixing of biobased resins and hardeners were carried out, choosing the best compromise in terms of mechanical properties and biobased content. Three resin formulations were identified to produce NFRC composites by a vacuum bagging process adding natural flax fibers (at 30 vol.%). The mechanical properties obtained for these new NFRC composites were promising, especially in terms of the biobased content that in one case reached 91%. A merit index for the new composites was calculated to give evidence as to how the stiffnesses are encouraging considering the light weight of the NFRC obtained. In fact, a merit index comparable to some typical epoxy–glass composites was obtained.

## Figures and Tables

**Table 1:** Resin name and composition.

Name	Epoxy Formulation Mix	Curing Agent Origin	Commercial Material Used	Ratio	Final Biobased Content (%)
MT1	Mix 50:50 Petro-based + Biobased	Petro-based	MIX 50:50 (EPIKOTE 828 + CARDOLITE NC-547) + SX8 EVOB	(100:30)	32.34
MB1	Mix 50:50 Biobased	Biobased	MIX 50:50 (CARDOLITE NC 547 + GREENPOXY S56) + CARDOLITE NC541	(100:68)	74.86
BB1	Biobased	Petro-based	GREENPOXY S56 + SD 4771	(100:30)	43.12

**Table 2:** Composites name and composition.

	Epoxy Resin	Fiber	Composite Name
Mix Petro-Bio based	[MIX 50:50 (EPIKOTE 828 + CARDOLITE NC-547)] + SX8 EVOB	Flax Twill	MT1-FT
Biobased	[MIX 50:50 (CARDOLITE NC 547 + GREENPOXY SR56)] + CARDOLITE NC541	Flax Twill	MB1-FT
Biobased	GREENPOXY S56 + SD 4771	Flax Twill	BB1-FT

**Table 3:** Results of tensile and DSC analysis on resin formulations.

ID	Biobased Content	Charpy Impact Strength (kJ/m^2^)	Tensile Tests			Tg (°C)
			E (GPa)	σ_b_ (MPa)	ε_b_ (%)	
MT1	32.34	Not detectable	0.77 ± 0.07	12.55 ± 0.97	6.89 ± 1.72	59.88 ± 0.01
MB1	74.86	Not detectable	0.45 ± 0.04	12.20 ± 1.02	6.45 ± 0.87	26.50 ± 0.01
BB1	43.12	6.02 ± 0.55	2.67 ± 0.42	45.66 ± 4.95	3.34 ± 1.76	52.30 ± 0.01

**Table 4:** Results of tensile tests, DSC, and HDT analysis on composites.

ID	Charpy Impact Strength (kJ/m^2^)	Tensile Tests			Tg (°C)
		E (GPa)	σ_b_ (MPa)	ε_b_ (%)	
MT1-FT	11.89 ± 0.15	4.69 ± 0.95	48.76 ± 5.64	2.25 ± 0.28	64.12 ± 0.01
MB1-FT	19.16 ± 1.69	2.69 ± 0.20	44.24 ± 3.27	7.41 ± 1.20	35.00 ± 0.01
BB1-FT	11.94 ± 2.07	6.34 ± 0.75	71.32 ± 6.06	2.72 ± 0.46	61.95 ± 0.01

## Data Availability

Data on request.

## References

[1] Andrew J.J., Dhakal H.N. (2022). Sustainable biobased composites for advanced applications: Recent trends and future opportunities—A critical review. Compos. Part C Open Access.

[2] Raponi E., Sergi C., Boria S., Tirillò J., Sarasini F., Calzolari A. (2021). Temperature effect on impact response of flax/epoxy laminates: Analytical, numerical and experimental results. Compos. Struct..

[3] Maiti S., Islam M.R., Uddin M.A., Afroj S., Eichhorn S.J., Karim N. (2022). Sustainable Fiber-Reinforced Composites: A Review. Adv. Sustain. Syst..

[4] La Mantia F.P., Morreale M. (2011). Green composites: A brief review. Compos. Part A Appl. Sci. Manuf..

[5] Joshi S., Drzal L., Mohanty A., Arora S. (2004). Are natural fiber composites environmentally superior to glass fiber reinforced composites?. Compos. Part A Appl. Sci. Manuf..

[6] Faruk O., Bledzki A.K., Fink H.P., Sain M. (2012). Biocomposites reinforced with natural fibers: 2000–2010. Prog. Polym. Sci..

[7] La Rosa A.D., Recca G., Summerscales J., Latteri A., Cozzo G., Cicala G. (2014). Bio-based versus traditional polymer composites. A life cycle assessment perspective. J. Clean. Prod..

[8] Fiore V., Scalici T., Di Bella G., Valenza A. (2015). A review on basalt fibre and its composites. Compos. Part B Eng..

[9] Chauhan V., Kärki T., Varis J. (2022). Review of natural fiber-reinforced engineering plastic composites, their applications in the transportation sector and processing techniques. J. Thermoplast. Compos. Mater..

[10] Sharma S., Sudhakara P., Nijjar S., Saini S., Singh G. (2018). Recent Progress of Composite Materials in various Novel Engineering Applications. Mater. Today Proc..

[11] Amiri A., Krosbakken T., Schoen W., Theisen D., Ulven C.A. (2017). Design and manufacturing of a hybrid flax/carbon fiber composite bicycle frame. Proc. Inst. Mech. Eng. Part P J. Sport. Eng. Technol..

[12] de Arcaya P.A., Retegi A., Arbelaiz A., Kenny J.M., Mondragon I. (2009). Mechanical properties of natural fibers/polyamides composites. Polym. Compos..

[13] Sarasini F., Fiore V. (2018). A systematic literature review on less common natural fibres and their biocomposites. J. Clean. Prod..

[14] Puglia D., Biagiotti J., Kenny J.M. (2005). A Review on Natural Fibre-Based Composites—Part II. J. Nat. Fibers.

[15] Oztoprak N., Gunes M.D., Tanoglu M., Aktas E., Egilmez O.O., Senocak C., Kulac G. (2018). Developing polymer composite-based leaf spring systems for automotive industry. Sci. Eng. Compos. Mater..

[16] Xia C., Yu J., Shi S.Q., Qiu Y., Cai L., Wu H.F., Ren H., Nie X., Zhang H. (2017). Natural fiber and aluminum sheet hybrid composites for high electromagnetic interference shielding performance. Compos. Part B Eng..

[17] Codispoti R., Oliveira D.V., Olivito R.S., Lourenço P.B., Fangueiro R. (2015). Mechanical performance of natural fiber-reinforced composites for the strengthening of masonry. Compos. Part B Eng..

[18] Dahy H. (2019). Natural Fibre-Reinforced Polymer Composites (NFRP) Fabricated from Lignocellulosic Fibres for Future Sustainable Architectural Applications, Case Studies: Segmented-Shell Construction, Acoustic Panels, and Furniture. Sensors.

[19] Thyavihalli Girijappa Y.G., Mavinkere Rangappa S., Parameswaranpillai J., Siengchin S. (2019). Natural Fibers as Sustainable and Renewable Resource for Development of Eco-Friendly Composites: A Comprehensive Review. Front. Mater..

[20] Bharath K.N., Madhu P., Gowda T.G.Y., Verma A., Sanjay M.R., Siengchin S. (2020). A novel approach for development of printed circuit board from biofiber based composites. Polym. Compos..

[21] Liu J., Zhang L., Shun W., Dai J., Peng Y., Liu X. (2021). Recent development on bio-based thermosetting resins. J. Polym. Sci..

[22] Witthayolankowit K., Rakkijakan T., Ayub R., Kumaniaev I., Pourchet S., Boni G., Watjanatepin P., Zarafshani H., Gabrion X., Chevallier A. (2023). Use of a fully biobased and non-reprotoxic epoxy polymer and woven hemp fabric to prepare environmentally friendly composite materials with excellent physical properties. Compos. Part B Eng..

[23] Verma A., Negi P., Singh V.K. (2019). Experimental Analysis on Carbon Residuum Transformed Epoxy Resin: Chicken Feather Fiber Hybrid Composite. Polym. Compos..

[24] Verma A., Baurai K., Sanjay M.R., Siengchin S. (2020). Mechanical, microstructural, and thermal characterization insights of pyrolyzed carbon black from waste tires reinforced epoxy nanocomposites for coating application. Polym. Compos..

[25] Marichelvam M.K., Manimaran P., Verma A., Sanjay M.R., Siengchin S., Kandakodeeswaran K., Geetha M. (2021). A novel palm sheath and sugarcane bagasse fiber based hybrid composites for automotive applications: An experimental approach. Polym. Compos..

[26] Crosky A., Soatthiyanon N., Ruys D., Meatherall S., Potter S., Hodzic A., Shanks R. (2014). Thermoset matrix natural fibre-reinforced composites. Natural Fibre Composites.

[27] Shundo A., Yamamoto S., Tanaka K. (2022). Network Formation and Physical Properties of Epoxy Resins for Future Practical Applications. JACS Au.

[28] Biswas E., Silva J.A., Khan M., Quirino R.L. (2022). Synthesis and Properties of Bio-Based Composites from Vegetable Oils and Starch. Coatings.

[29] Liu Z., Erhan S.Z., Akin D.E., Barton F.E. (2006). “Green” Composites from Renewable Resources:  Preparation of Epoxidized Soybean Oil and Flax Fiber Composites. J. Agric. Food Chem..

[30] Liu Z., Biswas A. (2013). Fluoroantimonic acid hexahydrate (HSbF6·6H2O) catalysis: The ring-opening polymerization of epoxidized soybean oil. Appl. Catal. A Gen..

[31] Park S.-J., Jin F.-L., Lee J.-R. (2004). Thermal and mechanical properties of tetrafunctional epoxy resin toughened with epoxidized soybean oil. Mater. Sci. Eng. A.

[32] Galdino D.S., Kondo M.Y., De Araujo V.A., Ferrufino G.L., Faustino E., Santos H.F., Christoforo A.L., Luna C.M., Campos C.I. (2023). Thermal and Gluing Properties of Phenol-Based Resin with Lignin for Potential Application in Structural Composites. Polymers.

[33] Caillol S. (2018). Cardanol: A promising building block for biobased polymers and additives. Curr. Opin. Green Sustain. Chem..

[34] Jaillet F., Darroman E., Ratsimihety A., Auvergne R., Boutevin B., Caillol S. (2014). New biobased epoxy materials from cardanol. Eur. J. Lipid Sci. Technol..

[35] Ng F., Couture G., Philippe C., Boutevin B., Caillol S. (2017). Bio-Based Aromatic Epoxy Monomers for Thermoset Materials. Molecules.

[36] Terry J.S., Taylor A.C. (2021). The properties and suitability of commercial bio-based epoxies for use in fiber-reinforced composites. J. Appl. Polym. Sci..

[37] Torres-Arellano M., Renteria-Rodríguez V., Franco-Urquiza E. (2020). Mechanical Properties of Natural-Fiber-Reinforced Biobased Epoxy Resins Manufactured by Resin Infusion Process. Polymers.

[38] Saidane E.H., Scida D., Ayad R. (2021). Thermo-mechanical behaviour of flax/green epoxy composites: Evaluation of thermal expansion coefficients and application to internal stress calculation. Ind. Crops Prod..

[39] Sala B., Gabrion X., Trivaudey F., Guicheret-Retel V., Placet V. (2021). Influence of the stress level and hygrothermal conditions on the creep/recovery behaviour of high-grade flax and hemp fibre reinforced GreenPoxy matrix composites. Compos. Part A Appl. Sci. Manuf..

[40] Bodros E., Pillin I., Montrelay N., Baley C. (2007). Could biopolymers reinforced by randomly scattered flax fibre be used in structural applications?. Compos. Sci. Technol..

[41] Sankar Lal S., Kannan S., Sahoo S.K. (2023). Investigation on the effect of castor-oil based bio-resins on mechanical, visco-elastic, and water diffusion properties of flax fiber reinforced epoxy composites. Polym. Compos..

[42] Biagiotti J., Puglia D., Torre L., Kenny J.M., Arbelaiz A., Cantero G., Marieta C., Llano-Ponte R., Mondragon I. (2004). A systematic investigation on the influence of the chemical treatment of natural fibers on the properties of their polymer matrix composites. Polym. Compos..

[43] Perremans D., Verpoest I., Dupont-Gillain C., Van Vuure A.W. (2018). Investigation of the tensile behavior of treated flax fibre bio-composites at ambient humidity. Compos. Sci. Technol..

[44] Wan Ramli W.M.A., Abdul Majid M.S., Ridzuan M.J.M., Sultan M.T.H., Amin N.A.M., Gibson A.G. (2020). The effect of nanomodified epoxy on the tensile and flexural properties of Napier fiber reinforced composites. Polym. Compos..

[45] Govignon Q., Bickerton S., Morris J., Kelly P.A. (2008). Full field monitoring of the resin flow and laminate properties during the resin infusion process. Compos. Part A Appl. Sci. Manuf..

[46] Muralidhara B., Kumaresh Babu S.P., Suresha B. (2020). Utilizing vacuum bagging process to prepare carbon fiber/epoxy composites with improved mechanical properties. Mater. Today Proc..

[47] Mustata A., Mustata F.S.C. (2013). Moisture Absorption and Desorption in Flax and Hemp Fibres and Yarns.

[48] Ali A., Shaker K., Nawab Y., Jabbar M., Hussain T., Militky J., Baheti V. (2016). Hydrophobic treatment of natural fibers and their composites—A review. J. Ind. Text..

[49] Sorrentino L., Turchetta S., Parodo G., Papa R., Toto E., Santonicola M.G., Laurenzi S. (2022). RIFT Process Analysis for the Production of Green Composites in Flax Fibers and Bio-Based Epoxy Resin. Materials.

[50] De Baere I., Van Paepegem W., Degrieck J. (2009). On the design of end tabs for quasi-static and fatigue testing of fibre-reinforced composites. Polym. Compos..

[51] Adams D.O., Adams D.F. (2002). Tabbing Guide for Composite Test Specimens.

[52] Yousif B.F. (2009). Frictional and wear performance of polyester composites based on coir fibres. Proc. Inst. Mech. Eng. Part J J. Eng. Tribol..

[53] Bachmann J., Wiedemann M., Wierach P. (2018). Flexural Mechanical Properties of Hybrid Epoxy Composites Reinforced with Nonwoven Made of Flax Fibres and Recycled Carbon Fibres. Aerospace.

[54] Rodrigues Junior S.A., Zanchi C.H., de Carvalho R.V., Demarco F.F. (2007). Flexural strength and modulus of elasticity of different types of resin-based composites. Braz. Oral Res..

[55] Zwawi M. (2021). A Review on Natural Fiber Bio-Composites, Surface Modifications and Applications. Molecules.

[56] Alsuwait R.B., Souiyah M., Momohjimoh I., Ganiyu S.A., Bakare A.O. (2022). Recent Development in the Processing, Properties, and Applications of Epoxy-Based Natural Fiber Polymer Biocomposites. Polymers.

[57] Fuqua M.A., Huo S., Ulven C.A. (2012). Natural Fiber Reinforced Composites. Polym. Rev..

[58] Le Guen M.J., Newman R.H. (2007). Pulped Phormium tenax leaf fibres as reinforcement for epoxy composites. Compos. Part A Appl. Sci. Manuf..

[59] Aliotta L., Lazzeri A. (2020). A proposal to modify the Kelly-Tyson equation to calculate the interfacial shear strength (IFSS) of composites with low aspect ratio fibers. Compos. Sci. Technol..

[60] Clyne T.W., Hull D. (2019). An Introduction to Composite Materials.

[61] Shercliff H.R., Ashby M.F. (2016). Elastic Structures in Design. Reference Module in Materials Science and Materials Engineering.

